# The safety profiles of avacopan on microscopic polyangiitis and granulomatosis with polyangiitis: a real-world pharmacovigilance analysis

**DOI:** 10.3389/fimmu.2025.1654735

**Published:** 2025-10-08

**Authors:** Yang Li, Anna Meng, Yue Wang

**Affiliations:** ^1^ Department of Pharmacy, Nanjing First Hospital, NanJing Medical University, Nanjing, China; ^2^ Department of Traditional Chinese Medicine, Nanjing First Hospital, Nanjing Medical University, Nanjing, China

**Keywords:** avacopan, adverse events, microscopic polyangiitis, granulomatosis with polyangiitis, FAERS, pharmacovigilance

## Abstract

**Background:**

Avacopan is an oral selective C5a receptor inhibitor. It received FDA approval in October 2021 for use with standard glucocorticoid therapy to treat antineutrophil cytoplasmic antibody-associated vasculitis. However, there are limitations to understanding adverse events (AEs) in clinical practice. In this study, we analyzed AEs related to avacopan by mining the FDA Adverse Event Reporting System (FAERS).

**Methods:**

Adverse event reports associated with avacopan were retrieved from the FAERS database covering Q1–2022 to Q4 2024. After data standardization, reports exclusively involving avacopan were retained. Two disproportionality analysis methods, namely the reporting odds ratio and Bayesian confidence propagation neural network, were utilized to detect safety signals related to avacopan. A semi-quantitative scoring approach was employed to evaluate the clinical priority of the detected signals. Moreover, racial disparities in the occurrence risks of critical adverse event signals as well as temporal patterns of avacopan-related AEs were examined.

**Results:**

Among 1,128 avacopan-related reports, 33 adverse event signals were identified. These included label-listed AEs (nausea, fatigue, diarrhea, headache, rash, hypertension, blood creatinine increased, and abnormal liver function). Five new potential AEs were detected, including alopecia, increased appetite, hyperaesthesia teeth, oesophageal disorder, and muscle atrophy. Clinically, 0 were classified as high-priority signals, 2 as moderate-priority signals, and 31 as low-priority signals. Compared to American patients, Japanese patients exhibited a higher risk of liver dysfunction (p<0.001), while alopecia occurred exclusively in the American patient population. The median time to AE onset time for avacopan was 86.5 days(interquartile range [IQR] 27-236.75), with most occurring within the first month of treatment.

**Conclusions:**

Avacopan exhibits favorable real-world safety with no high-priority AEs identified. Our findings may provide important evidence for future clinical research and the management of safety issues related to avacopan.

## 1 Introduction

Anti-neutrophil cytoplasmic antibody (ANCA)-associated vasculitis (AAV) is a group of systemic autoimmune diseases characterized by small vessel vasculitis, with complex pathogenesis and a high rate of relapse (1). Classic ANCA-associated vasculitis includes granulomatosis with polyangiitis, microscopic polyangiitis, and eosinophilic granulomatosis with polyangiitis. Recent ultrastructural studies have shown that ANCA and complement mediate early vascular lesions in vasculitis through neutrophil infiltration and endothelial cell changes, providing a morphological basis for targeted therapy (2). Conventional therapies for ANCA-associated vasculitis primarily include glucocorticoids and immunosuppressants, which are plagued by high relapse rates and frequent adverse reactions (3, 4).

Avacopan is a complement C5a receptor (C5aR) antagonist that received FDA approval in October 2021 for the treatment of AAV and other autoimmune disorders (5, 6). It functions therapeutically by disrupting the interaction between C5a and its receptor, thereby inhibiting C5a-mediated neutrophil activation and migration. Studies indicate that patients receiving avacopan treatment exhibit superior safety and improved quality of life compared to AAV patients treated with glucocorticoids (7–10). Subsequently, the 2022 management recommendations for AAV and the 2023 guidelines from the Ministry of Health, Labour and Welfare of Japan successively recommended avacopan as a substitute for glucocorticoids in remission induction regimens (11, 12). Clinical trials are often limited by restricted patient enrollment and short follow-up durations (13, 14). Currently, there are few post-marketing safety studies of avacopan based on real-world data. Consequently, we assessed the post-marketing safety of avacopan using pharmacovigilance analysis of the FAERS database, with the goal of offering insights for healthcare professionals in ensuring the safe use of avacopan.

## 2 Methods

### 2.1 Data source and processing

This study used data from the FAERS database. AE reports identifying avacopan (generic name) and Tavneos (brand name) as the primary suspected drug were collected, spanning Q1–2022 to Q4 2024. Before analysis, deduplication was performed following FDA guidelines: for reports sharing the same CASEID, those with the largest FDA_DT value were preserved; if both CASEID and FDA_DT were identical, the report with the highest PRIMARYID value was retained. Next, reports of AEs related exclusively to avacopan were filtered from the DRUG table using the ROLE_COD field. Additionally, AEs in the FAERS database were encoded using preferred terms (PTs) and categorized into system organ classes (SOCs) based on the Medical Dictionary for Regulatory Activities (MedDRA v27.1).

### 2.2 Statistical analysis

Two disproportionality analysis methods were employed to detect adverse event signals related to the drug: the reporting odds ratio (ROR) and Bayesian confidence propagation neural network (BCPNN). An AE was considered to yield a signal if it met the positive criteria of both methods. Relevant formulas and thresholds were provided in **Supplementary Table 1**. Additionally, the Chi-square test was used to compare the risk of target AEs across different racial groups, with p<0.05 considered statistically significant. Data processing and statistical analyses were performed using RStudio (v4.3.2) and Microsoft Excel 2019.

### 2.3 Clinical prioritization of signals

To identify suspicious adverse drug reaction reports that require special attention and prioritize safety reviews, the European Medicines Agency (EMA) has developed and updated lists of Important Medical Events (IME) and Designated Medical Events (DME). We assessed and ranked significant disproportionate AEs by evaluating five different characteristics to determine their clinical priority: number of reports, ROR025, proportion of deaths, designation of IMEs or DMEs, and evidence evaluation. Three levels of clinical significance were used for prioritization, including weak, moderate, or strong, with semi-quantitative scoring ranging from 0-4, 5-7, or 8-10, as detailed in **Supplementary Table 2**.

### 2.4 Time to onset analysis

The time to onset of adverse reactions was defined as the interval between the initiation of avacopan use and the date of AE occurrence. Prior to analysis, we de-duplicated the data and removed missing data. The median interquartile range (IQR) was used in this study to evaluate the characteristics of adverse reaction occurrence.

## 3 Results

### 3.1 Descriptive analysis

**Figure 1:**
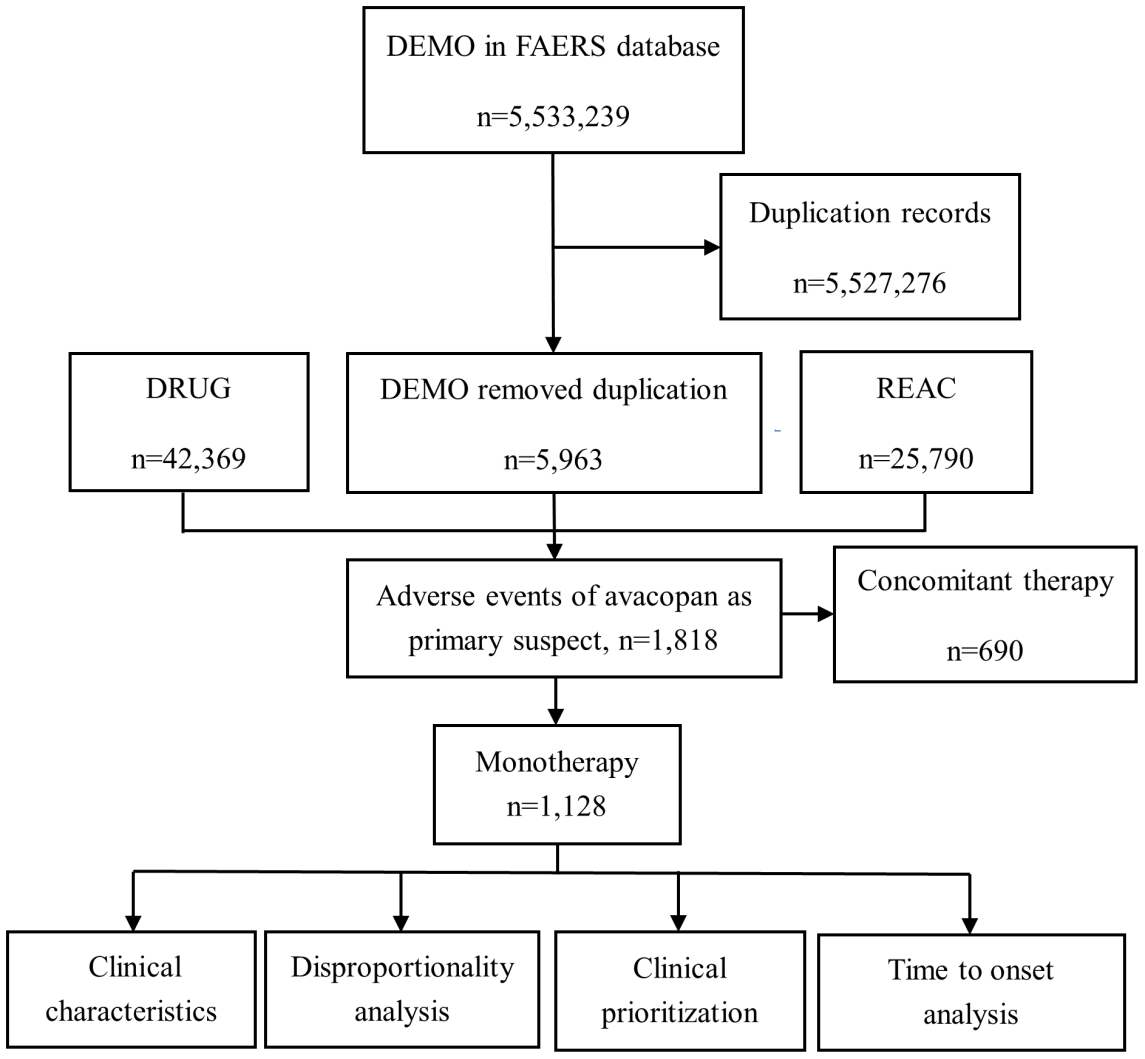
Flowchart of adverse event screening for Avacopan in the FAERS database. This diagram illustrates the workflow for identifying, cleaning, and analyzing adverse event reports related to avacopan using the FAERS database. DEMO, Patient demographic and administrative information file; DRUG, Drug information, including the primary suspect drug, secondary suspect drugs, concomitant medications, or interacting drugs; REAC, Adverse event report file; N, number.

Among the 2,265 adverse event reports collected from the FAERS database, 1,128 were related to avacopan, and the screening process is shown in **Figure 1**.

**Table 1** outlines the clinical characteristics of these adverse event reports. By gender, female patients accounted for 47.0% vs. 28.4% in males. The most frequent age group was 45–64 years (29.2%), followed by ≥65 years (27.5%). Geographically, the United States contributed the largest share of reports (89.3%), followed by Japan (5.1%) and Canada (2.7%). Severe outcomes related to AEs were highest for hospitalization or prolonged hospital stay, accounting for 19.5%, followed by death, accounting for 6.9%.

**Table 1:** Baseline characteristics of adverse events related to Avacopan.

Characteristics	Case, n	Percentage, %
Number of events	1,128	100
Gender		
Female	530	47.0
Male	320	28.4
Missing	278	24.6
Age		
<18	13	1.2
18~45	151	13.4
45~65	329	29.2
≥65	310	27.5
Unknown	325	28.8
Indications		
Anti-neutrophil cytoplasmic antibody positive vasculitis	286	25.4
Granulomatosis with polyangiitis	618	54.8
Microscopic polyangiitis	224	19.8
Serious outcome		
Hospitalization-initial or prolonged	220	19.5
Death	78	6.9
Life-threatening	5	0.4
Disabled	2	0.2
Other important medical event	823	73.0
Reported countries (Top three)		
United States	1007	89.3
Japan	57	5.1
Canada	31	2.7

### 3.2 Signal detection

AEs associated with avacopan occurred across 24 System Organ Classes (SOCs). Six SOC levels yielded valid signals. The top three SOC was gastrointestinal disorders, followed by surgical and medical procedures and infections and infestations, as detailed in **Table 2**.

**Table 2:** Signal strength of reports of avacopan at the System Organ Class (SOC) level in FAERS database.

SOC	Cases, n	ROR (95%CI)	IC (IC025)
General disorders and administration site conditions	421	1.08 (0.97 - 1.2)	0.09 ( -0.06)
Gastrointestinal disorders*	296	1.62 (1.43 - 1.83)	0.62 (0.44)
Surgical and medical procedures*	204	7.16 (6.2 - 8.27)	2.72 (2.47)
Infections and infestations*	186	1.61 (1.38 - 1.87)	0.64 (0.41)
Injury, poisoning and procedural complications	185	0.76 (0.65 - 0.88)	-0.36 ( -0.58)
Nervous system disorders	152	0.78 (0.66 - 0.92)	-0.33 (-0.57)
Investigations	140	1.01 (0.85 - 1.2)	0.01 (-0.24)
Respiratory, thoracic and mediastinal disorders	119	1.12 (0.93 - 1.35)	0.16 (-0.11)
Skin and subcutaneous tissue disorders	106	0.86 (0.7 - 1.04)	-0.21 (-0.5)
Musculoskeletal and connective tissue disorders	93	0.78 (0.64 - 0.96)	-0.34 (-0.64)
Vascular disorders*	63	1.32 (1.03 - 1.69)	0.39 (0.02)
Renal and urinary disorders	56	1.32 (1.01 - 1.72)	0.39 (-0.01)
Psychiatric disorders	46	0.35 (0.26 - 0.47)	-1.46 (-1.86)
Hepatobiliary disorders*	43	2.08 (1.54 - 2.81)	1.04 (0.57)
Eye disorders	32	0.7 (0.5 – 1.0)	-0.5 (-0.99)
Metabolism and nutrition disorders	31	0.63 (0.44 - 0.9)	-0.66 (-1.16)
Cardiac disorders	26	0.43 (0.3 - 0.64)	-1.18 (-1.71)
Immune system disorders	22	0.87 (0.57 - 1.33)	-0.19 (-0.79)
Ear and labyrinth disorders*	17	1.74 (1.08 - 2.81)	0.8 (0.05)
Blood and lymphatic system disorders	11	0.28 (0.16 - 0.51)	-1.8 (-2.55)
Product issues	7	0.18 (0.09 - 0.39)	-2.42 (-3.29)
Social circumstances	4	0.37 (0.14 – 1.0)	-1.41 (-2.51)
Neoplasms benign, malignant and unspecified (incl cysts and polyps)	3	0.05 (0.02 - 0.15)	-4.29 (-5.34)
Endocrine disorders	2	0.34 (0.09 - 1.37)	-1.54 (-2.85)

*indicates statistically significant signals in algorithm.

Using two algorithms, we identified 33 AEs associated with avacopan as valid signals (**Table 3**). The AEs identified included nausea, fatigue, diarrhea, headache, rash, and upper abdominal pain. These findings align with the drug label. This study found that the incidence of liver dysfunction-related AEs in East Asian populations was significantly higher than that in Caucasian populations (p < 0.001). In contrast, reports of alopecia as an AE came solely from Caucasian populations. Additionally, 5 new potential AEs, not listed on the drug label, such as hair loss, increased appetite, muscle atrophy, oesophageal disorder, and hyperaesthesia teeth, have also been observed.

**Table 3:** Signal strength of reports of avacopan at the PT level in FAERS database.

PT	Cases, n	ROR (95%CI)	IC (IC025)
Nausea	65	2.31 (1.80 - 2.96)	1.18 (0.8)
Fatigue	65	2.34 (1.83 – 3.0)	1.2 (0.81)
Diarrhoea	61	2.68 (2.08 - 3.46)	1.4 (0.99)
Headache	39	1.72 (1.25 - 2.36)	0.77 (0.28)
Rash	29	1.76(1.22-2.55)	0.81 (0.24)
Abdominal Discomfort	27	4.36 (2.99 - 6.38)	2.11 (1.4)
Hypertension	24	3.12 (2.08 - 4.66)	1.63 (0.93)
Alopecia*	23	3.2 (2.12 - 4.83)	1.67 (0.95)
Pneumonia	23	1.85 (1.23 - 2.79)	0.88 (0.24)
Infection	22	4.27 (2.8 - 6.5)	2.08 (1.29)
Upper abdominal pain	22	2.99 (1.96 - 4.54)	1.57 (0.84)
Cough	21	2.08 (1.36 - 3.2)	1.05 (0.37)
Swelling face	18	7.64 (4.81 - 12.16)	2.92 (1.83)
Blood pressure increased	18	3.22 (2.02 - 5.11)	1.68 (0.85)
Hepatic enzyme increased	18	7.41 (4.66 - 11.78)	2.88 (1.80)
Peripheral swelling	16	2.92 (1.78 - 4.77)	1.54 (0.68)
Liver disorder	10	6.23 (3.35 - 11.6)	2.63 (1.20)
Hepatic function abnormal	10	7.52 (4.04 - 13.99)	2.9 (1.36)
Swelling	9	2.22 (1.15 - 4.28)	1.15 (0.07)
Blood creatinine increased	8	3.3 (1.65 - 6.62)	1.72 (0.43)
Liver function test increased	8	10.75 (5.37 - 21.52)	3.42 (1.40)
Drug-induced liver injury	8	7.66 (3.83 - 15.34)	2.93 (1.17)
Aspartate aminotransferase increased	7	3.57 (1.70 - 7.49)	1.83 (0.41)
Alanine aminotransferase increased	7	3.09 (1.47 - 6.48)	1.62 (0.27)
Pollakiuria	6	3.97 (1.78 - 8.85)	1.99 (0.38)
Increased appetite*	5	7.86 (3.27 - 18.9)	2.97 (0.69)
Blood alkaline phosphatase increased	5	5.35 (2.22 - 12.86)	2.42 (0.45)
Blood pressure abnormal	4	5.66 (2.12 - 15.1)	2.5 (0.26)
Localised infection	4	4.52 (1.70 - 12.07)	2.18 (0.11)
Muscle atrophy*	3	6.72 (2.17 - 20.86)	2.75 (0.02)
Oesophageal disorder*	3	20.96 (6.75 - 65.07)	4.39 (0.36)
Pharyngeal swelling	3	9.21 (2.97 - 28.59)	3.2 (0.15)
Hyperaesthesia teeth*	3	27.47 (8.85 - 85.28)	4.78 (0.41)

*Emerging findings of avacopan-associated adverse events from the FAERS database.

### 3.3 Clinical prioritization assessment of signals

Summary information on AE reporting cases, signal strength, mortality, IME, DME, and relevant evidence assessments associated with avacopan is compiled in **Table 4**. Overall, among the 33 preferred terms, pneumonia (3.03%) was classified as important medical event, while drug-induced liver injury (3.03%) was classified as designated medical events. According to the clinical priority scoring criteria, no AEs of high clinical priority were identified. Drug-induced liver injury and diarrhea are medium-intensity risk signals, while the remaining 31 risk signals are all of low clinical priority.

**Table 4:** Signal strength of the Preferred Term (PT) and the clinical priority assessing results.

PT	Case, n	ROR (95%CI)	IC (IC025)	Death, n	IME/ DME	Relevant evidence evaluation	Priority level(score)
Nausea	65	2.31 (1.80 - 2.96)	1.18 (0.8)	0	NA	**++**	Weak (4)
Fatigue	65	2.34 (1.83 – 3.0)	1.2 (0.81)	0	NA	**++**	Weak (4)
Diarrhoea	61	2.68 (2.08 - 3.46)	1.4 (0.99)	0	NA	**++**	Moderate (5)
Headache	39	1.72 (1.25 - 2.36)	0.77 (0.28)	1	NA	**++**	Weak (3)
Rash	29	1.76(1.22-2.55)	0.81(0.24)	0	NA	**++**	Weak (3)
Abdominal discomfort	27	4.36 (2.99 - 6.38)	2.11 (1.4)	0	NA	**++**	Weak (4)
Hypertension	24	3.12 (2.08 - 4.66)	1.63 (0.93)	0	NA	**++**	Weak (4)
Alopecia*	23	3.2 (2.12 – 4.83)	1.67 (0.95)	0	NA	–	Weak (2)
Pneumonia	23	1.85 (1.23 - 2.79)	0.88 (0.24)	4	IME	**++**	Weak (4)
Infection	22	4.27 (2.8 - 6.5)	2.08 (1.29)	3	NA	**++**	Weak (4)
Upper abdominal pain	22	2.99 (1.96 - 4.54)	1.57 (0.84)	0	NA	**++**	Weak (3)
Cough	21	2.08 (1.36 - 3.2)	1.05 (0.37)	0	NA	**+**	Weak (2)
Swelling face	18	7.64 (4.81 - 12.16)	2.92 (1.83)	0	NA	**++**	Weak (4)
Blood pressure increased	18	3.22 (2.02 - 5.11)	1.68 (0.85)	0	NA	**++**	Weak (4)
Hepatic enzyme increased	18	7.41 (4.66 - 11.78)	2.88 (1.80)	0	NA	**++**	Weak (4)
Peripheral swelling	16	2.92 (1.78 - 4.77)	1.54 (0.68)	0	NA	**++**	Weak (3)
Liver disorder	10	6.23 (3.35 - 11.6)	2.63 (1.20)	1	NA	**++**	Weak (4)
Hepatic function abnormal	10	7.52 (4.04 - 13.99)	2.9 (1.36)	0	NA	**++**	Weak (4)
Swelling	9	2.22 (1.15 - 4.28)	1.15 (0.07)	0	NA	**++**	Weak (2)
Blood creatinine increased	8	3.3 (1.65 - 6.62)	1.72 (0.43)	0	NA	**++**	Weak (2)
Liver function test increased	8	10.75 (5.37-21.52)	3.42 1.40)	0	NA	**++**	Weak (4)
Drug-induced liver injury	8	7.66 (3.83 - 15.34)	2.93 (1.17)	0	DME	**++**	Moderate (5)
Aspartate aminotransferase increased	7	3.57 (1.70 - 7.49)	1.83 (0.41)	0	NA	**++**	Weak (2)
Alanine aminotransferase increased	7	3.09 (1.47 - 6.48)	1.62 (0.27)	0	NA	**++**	Weak (2)
Pollakiuria	6	3.97 (1.78 - 8.85)	1.99 (0.38)	0	NA	**++**	Weak (2)
Increased appetite*	5	7.86 (3.27 - 18.9)	2.97 (0.69)	0	NA	–	Weak (1)
Blood alkaline phosphatase increased	5	5.35 (2.22 - 12.86)	2.42 (0.45)	0	NA	**++**	Weak (3)
Blood pressure abnormal	4	5.66 (2.12 - 15.1)	2.5 (0.26)	0	NA	**++**	Weak (3)
Localised infection	4	4.52 (1.70 - 12.07)	2.18 (0.11)	0	NA	**++**	Weak (2)
Muscle atrophy*	3	6.72 (2.17 - 20.86)	2.75 (0.02)	0	NA	–	Weak (1)
Oesophageal disorder*	3	20.96 (6.75-65.07)	4.39 (0.36)	0	NA	–	Weak (2)
Pharyngeal swelling	3	9.21 (2.97 - 28.59)	3.2 (0.15)	0	NA	**++**	Weak (3)
Hyperaesthesia teeth*	3	27.47 (8.85-85.28)	4.78 (0.41)	0	NA	–	Weak (2)

CI, Confidence Interval; DME, Designated Medical Event; IC, Information Component; IME, Important Medical Event; NA, Not applicable; PT, Preferred Term; ROR, Reporting Odds Ratio; n, Number of cases. ++: AEs are mainly from the FDA Prescribing Information, the Summary of Product Characteristics of avacopan posted by the MHRA, Phase 2/3 RCTs, or systematic reviews, with biological plausibility. +: AEs are mainly from other clinical trials, observational studies, or case reports/series with potential biological plausibility. -: AEs only emerge from disproportionality analyses. *: AEs not recorded in the drug label.

### 3.4 Time to onset of avacopan-related AEs

**Figure 2:**
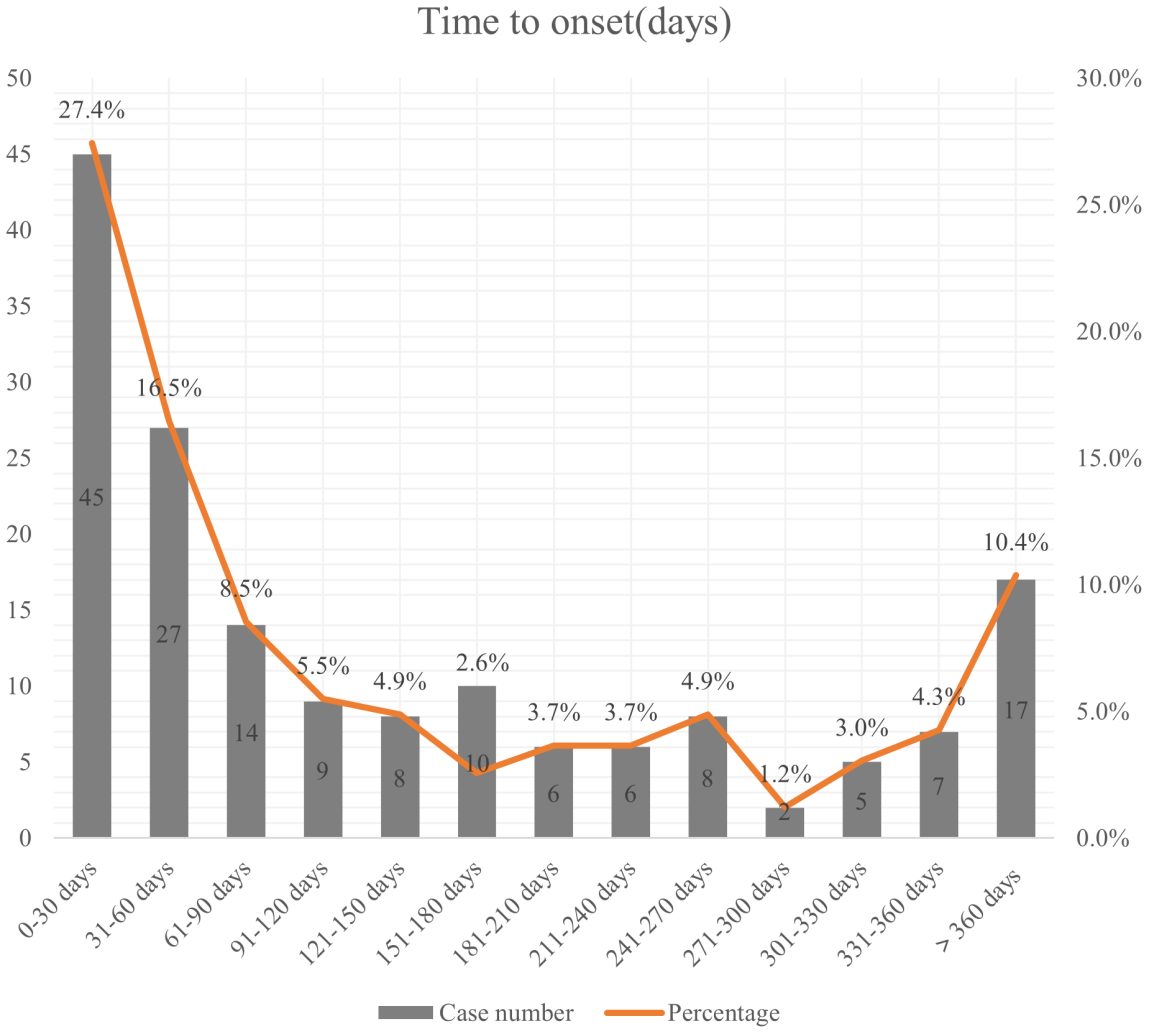
Time to onset of avacopan-related adverse events.

164 patients reported the occurrence time of AEs. The median onset time was 86.5 days (IQR 27-236.75). As shown in **Figure 2**, the occurrence of adverse reactions was most common within the first month after medication initiation, accounting for 27.4%. Approximately 10.4% of adverse reactions occurred after 1 year or longer of treatment.

## 4 Discussion

To our knowledge, this study is the first to conduct a comprehensive evaluation of the safety profile of avacopan using the FAERS database. It identified common AEs listed in the drug label and uncovered new potential AEs. Additionally, all AEs were ranked based on their clinical priorities. This study is expected to provide valuable references for the rational use of avacopan in clinical practice.

Epidemiologic research demonstrates that the prevalence of AAV is marginally higher among males as compared to females (15). In this study, the number of females in the reporting population exceeded that of males, which may be attributed to the higher proportion of missing gender data in the reporting population. Furthermore, this might be linked to the lack of availability of data regarding the numbers of male and female patients prescribed avacopan. Research indicates that the average age of patients with GPA is 57 ± 5.74 years, while the average age of patients with MPA is 65 ± 5.89 years (16). This study found that the age group of 45 to 65 years comprised the largest proportion, which is hypothesized to be associated with the higher percentage of GPA patients in this research. The United States reported the highest number of AEs, which may be related to the initial market launch of avacopan in the U.S.

In this study, we observed a greater frequency of AE signals related to avacopan treatment, such as nausea, fatigue, diarrhea, headache, rash, and upper abdominal pain, which align with the common side effects documented in the drug label. In addition, our study also identified hepatotoxic adverse reactions mentioned in the warnings and precautions section of the drug label, including elevated liver enzymes, liver disorder, and drug-induced liver injury. Research has reported that treatment with avacopan can lead to liver function impairment (17–20) Although no dose adjustment of avacopan is required for patients with mild to moderate liver dysfunction, this AE should not be overlooked (6). A retrospective study by Genri Tagami et al. found that elevated liver enzyme levels are the most common adverse reaction associated with avacopan treatment (21). Another study found that the most common reason for discontinuation of avacopan was abnormal serum transaminase levels (22). Currently, the mechanism by which avacopan causes liver injury is unclear, but it may be idiosyncratic and immune-mediated (23). Our study found that the risk of liver dysfunction in the Japanese population is significantly higher than that in the American population (p<0.001). According to literature reports, liver function impairment AEs resolved after discontinuation of avacopan and other potentially hepatotoxic drugs. The study by Hiroshi Kataoka et al. found that gradually increasing the dose of avacopan in combination with the use of ursodeoxycholic acid may also help avoid the risk of liver injury induced by avacopan (24). A study conducted in Japan indicates that the risk factors for the development of severe drug induced liver injury (DILI) with elevated T-Bil in patients treated with avacopan include advanced age, lower body mass index, and early onset of DILI after the initiation of avacopan therapy (25). Therefore, for patients undergoing avacopan treatment, particularly the elderly and those with a low body mass index, enhanced monitoring of liver function and recognition of liver-related clinical symptoms should be prioritized.

Serious infections are similarly included in the warnings and precautions section of the avacopan drug label. Research indicates that infections are the leading cause of mortality in patients with AAV during the first year following the initiation of treatment (26). Our research identified AE signals associated with infections, including localized infections and infectious pneumonia. In the ADVOCATE III clinical trial, approximately two-thirds of patients in the avacopan group experienced infectious AEs, of which 10.3% were classified as severe (27). In another multicenter, retrospective cohort study spanning more than 52 weeks, infections were the most frequently reported AEs, with approximately one-third of patients experiencing infections that required hospitalization (28). Therefore, considering the incidence and severity of infections, it is important to enhance monitoring for infection-related symptoms and signs at the initiation of therapy. If a serious active infection develops, the medication should be discontinued until the infection is resolved.

Hypersensitivity reactions are one of the adverse effects reported in the clinical trials of avacopan (29, 30). This study identified AEs such as facial swelling, pharyngeal swelling, and peripheral oedema, all of which are clinical manifestations of allergic reactions. Given that angioedema can sometimes lead to serious consequences, patients must promptly notify their physician if they experience symptoms such as swelling of the face, lips, or tongue while taking avacopan.

This study also identified new potential AEs, one of which was alopecia. There have been no previous reports, and it is not listed in the product labeling. Although this study utilized two methods to uncover potential AEs, the AE of alopecia met the criteria of all four signal calculation methods: reporting odds ratio, proportional reporting ratio, gamma-Poisson shrinkage method, and Bayesian confidence propagation neural network. Antibodies targeting the C5 precursor C5(eculizumab) and C5a(vilobelimab) have received FDA approval. Specifically, one study documented alopecia as an AE resulting from treatment with eculizumab (31). Given the similarity in the mechanisms of action between avacopan and eculizumab, it is recommended to strengthen monitoring in patients undergoing avacopan therapy. It is noteworthy that all reported cases of alopecia as AEs were observed in patients from the Americas, with no Asian patients affected. This discrepancy may suggest a potential link to ethnicity. The precise mechanism underlying the alopecia AE caused by avacopan needs to be confirmed through future clinical practice or clinical studies. Moreover, our study uncovered additional new potential AEs such as oesophageal disorder, muscle atrophy, hyperaesthesia teeth, and increased appetite, which were all categorized as low clinical priority. Clinicians should be cautious about these potential new AEs and intensify patient monitoring during the drug administration period.

It is worth mentioning that, although no effective signals were generated, this study identified four AEs related to drug interactions. Given that avacopan is both an inhibitor and substrate of CYP3A4, vigilance regarding drug interactions is essential during treatment (32). The study found that avacopan can increase the area under the curve of co-administered CYP3A4 or CYP2C9 substrates, indicating a potential interaction (33). Additionally, avacopan has a half-life of up to 21 days, so potential drug interactions should be considered not only during treatment but also for several weeks after discontinuation of avacopan, in order to maximize the safety of patient treatment.

The median time to onset of AEs linked to avacopan was identified as 86.5 days in our findings. Most AEs were reported within the first month after the initiation of avacopan treatment. Moreover, a notable proportion of AEs occurred after one year of avacopan treatment, suggesting the need for both initial close monitoring and extended patient surveillance over the course of therapy. In clinical practice, greater emphasis should be placed on the early identification and management of AEs related to avacopan.

Several limitations of the present study should be emphasized. Firstly, the spontaneous and open-access nature of the FAERS database inherently carries risks of underreporting and misreporting. Secondly, multiple confounding factors including reporter identity, disease severity, ethnicity, and demographic characteristics of medication users may have influenced the observed results. Thirdly, disproportionality analysis merely indicates a statistical association between avacopan and AEs, rather than establishing a definitive causal relationship; thus, these findings should be interpreted as exploratory signal detection analyses. Furthermore, the overwhelming majority of reports originating from the United States may introduce potential geographical bias, leading to underestimation of region-specific risks in Asian populations. Despite these limitations, our study identifies novel avacopan-associated AEs and provides critical insights for its clinical safety evaluation.

## 5 Conclusion

Through the identification of AEs related to avacopan and their classification based on clinical importance, our study may offer important insights for the future research and clinical safety management of avacopan.

## Data Availability

The original contributions presented in the study are included in the article/**Supplementary Material**. Further inquiries can be directed to the corresponding author.
