# Supplementary material for: The safety profiles of avacopan on microscopic polyangiitis and granulomatosis with polyangiitis: a real-world pharmacovigilance analysis
Source: Front Immunol. 2025 Oct 8;16:1654735. doi: 10.3389/fimmu.2025.1654735 (PMC12540178; doi:10.3389/fimmu.2025.1654735)
Supplement: Supplementary file 1 [file Table1.docx]

**Supplementary Table 1**. Formulas and criteria for signal detection algorithms

| **Algorithms** | **Equation** | **Criteria** |
| --- | --- | --- |
| ROR | ROR = ad/bc | a≥3, lower limit of 95% CI > 1 |
|  | 95% CI = eln ^(ROR)±1.96 (1/a+1/b+1/c+1/d)^0.5^ |  |
| BCPNN | IC = log_2_^a (a+b + c + d) (a+c) (a+b)^ | a≥3, IC-2SD > 0 |
|  | 95% CI = E (IC) ± 2 V(IC)^^0.5^ |  |

Note: ROR: reporting odds ratio; BCPNN: bayesian confidence propagation neural network; a:represents the number of targeted adverse events for the target drug; b: represents the number of other adverse events for the target drug; c:represents the number of targeted adverse events for other drugs; d: represents the number of other adverse events for other drugs; CI: confidence interval; IC: information component; IC-2SD: lower limit of 95% CI of the IC; E(IC): the expected value of IC; V(IC): the variance of IC.
