# Supplementary material for: The safety profiles of avacopan on microscopic polyangiitis and granulomatosis with polyangiitis: a real-world pharmacovigilance analysis
Source: Front Immunol. 2025 Oct 8;16:1654735. doi: 10.3389/fimmu.2025.1654735 (PMC12540178; doi:10.3389/fimmu.2025.1654735)
Supplement: Supplementary file 2 [file Table2.docx]

**Supplementary Table 2**. A rating scale assessing clinical priority of disproportionality signals

| **Assessment items** | **2 points** | **1 point** | **0 point** |
| --- | --- | --- | --- |
| Number of target events | >50 | 10-50 | <10 |
| RORL | >5 | 2-5 | 1-2 |
| Mortality proportion | >50% | 25-50% | <25% |
| IMEs or DMEs | DME | IME | None |
| Relevant evidence evaluation | ++ | + | **-** |

Note: Mortality proportion: percentage of cases in which death was reported as an outcome in the overall cases report for a particular adverse event. ++: AEs are mainly from the FDA Prescribing Information, the Summary of Product Characteristics of avacopan posted by the MHRA, Phase 2/3 RCTs, or systematic reviews, with biological plausibility. +: AEs are mainly from other clinical trials, observational studies, or case reports/series with potential biological plausibility. -: AEs only emerging from disproportionality analyses. DMEs: Designated Medical Events; IMEs: Important Medical Events.
